# Social vulnerability and surgery outcomes: a cross-sectional analysis

**DOI:** 10.1186/s12889-024-19418-5

**Published:** 2024-07-16

**Authors:** Mohamed Abdelhack, Sandhya Tripathi, Yixin Chen, Michael S. Avidan, Christopher R. King

**Affiliations:** 1 Department of Anesthesiology, Washington University School of Medicine, St. Louis, MO USA; 2 Department of Computer Science, Washington University in St. Louis, St. Louis, MO USA; 3 Krembil Centre for Neuroinformatics, Centre for Addiction and Mental Health, Toronto, ON Canada

**Keywords:** Social vulnerability, Postsurgical complications, Housing status, Socioeconomic status, Sex interaction

## Abstract

**Background:**

Post-operative complications present a challenge to the healthcare system due to the high unpredictability of their incidence. Socioeconomic conditions have been established as social determinants of health. However, their contribution relating to postoperative complications is still unclear as it can be heterogeneous based on community, type of surgical services, and sex and gender. Uncovering these relations can enable improved public health policy to reduce such complications.

**Methods:**

In this study, we conducted a large population cross-sectional analysis of social vulnerability and the odds of various post-surgical complications. We collected electronic health records data from over 50,000 surgeries that happened between 2012 and 2018 at a quaternary health center in St. Louis, Missouri, United States and the corresponding zip code of the patients. We built statistical logistic regression models of postsurgical complications with the social vulnerability index of the tract consisting of the zip codes of the patient as the independent variable along with sex and race interaction.

**Results:**

Our sample from the St. Louis area exhibited high variance in social vulnerability with notable rapid increase in vulnerability from the south west to the north of the Mississippi river indicating high levels of inequality. Our sample had more females than males, and females had slightly higher social vulnerability index. Postoperative complication incidence ranged from 0.75% to 41% with lower incidence rate among females. We found that social vulnerability was associated with abnormal heart rhythm with socioeconomic status and housing status being the main association factors. We also found associations of the interaction of social vulnerability and female sex with an increase in odds of heart attack and surgical wound infection. Those associations disappeared when controlling for general health and comorbidities.

**Conclusions:**

Our results indicate that social vulnerability measures such as socioeconomic status and housing conditions could affect postsurgical outcomes through preoperative health. This suggests that the domains of preventive medicine and public health should place social vulnerability as a priority to achieve better health outcomes of surgical interventions.

**Supplementary Information:**

The online version contains supplementary material available at 10.1186/s12889-024-19418-5.

## Introduction

Post-operative complications are adverse events that could occur after a surgical procedure. Existing international studies report one in six patients undergoing elective surgery have at least one postoperative complication before discharge [1–3]. Postoperative complications can vary in prevalence (infectious complications 9%, cardiovascular complications 4.5%, kidney injury 1.7%) based on the surgery type. The impact of these complications can be seen in the reduction of life expectancy and quality of life, increase in treatment costs, the need for critical care, in-hospital mortality and even complication led readmission. The prevalence of this rehospitalization is notably elevated, standing at 4.7% and 2.87% following general surgery in the United Kingdom [4] and South Africa [5] and reaching 15.1% after colorectal cancer surgery among a substantially large patient cohort in New Zealand [6]. Multiple factors impact postoperative complications. Patient and hospital-level factors have been well studied, but understanding the social determinants of health (SDOH) for patient outcomes has become an important area of research due to the surfacing of disparities among SDOH [7–9]. For example, medical causes (such as circulatory or respiratory complications) of postoperative readmission were found to be twice as common in older patients in an age (SDOH) stratified analysis [10].

Research on SDOH faces challenges due to limited, accurate data in electronic health records (EHR) and administrative records. Racial disparities in surgical outcomes are often examined due to race being routinely recorded [11]. US Census and survey derived freely available CDC's Social Vulnerability Index (SVI) combines economic, personal, social, and ethnic factors into a single metric, originally intended for disaster resilience assessment [12]. Derived from 16 variables, the census tract level SVI is categorized into four themes: socioeconomic status, household composition & disability, minority status & language, and housing type & transportation.

The relationship between SVI and health outcomes has been explored in several existing works. Before SVI, median household income of the residential zip code has been a proxy for SES with a previous study observing that out of the patients admitted to hospital for acute pulmonary embolism, patients from lower median-income neighborhoods had a higher incidence of mortality compared to patients residing in higher SES zip codes [13]. CDC’s SVI has been correlated to worse surgical outcomes: 1) In cancer surgeries [14] about one-half of the Medicare beneficiaries experienced either a complication or extended length of stay or were readmitted within 90-days along with SVI being associated with the adverse outcomes, 2) In hepatopancreatic surgeries [16] a cluster analysis on the SVI subthemes showed the association between all vulnerability groups and outcomes in Medicare beneficiaries, 3) In Medicare patients undergoing colon resection the risk of postoperative pulmonary failure and pneumonia was observed to be higher [16], 4) In colectomy [17] and esophagectomy [19] patients even after adjusting for baseline demographics, higher SVI was associated with increased risk of postoperative complications. Social vulnerability (to an event in health, nature or society) has been found to be associated with gender and sex where female-headed households were most vulnerable [19–21]. Previous work has studied the postsurgical complications in females and women and their relation to SVI but they fell short of comparing the additional risk that is associated with sex and gender [22]. There have been multiple studies in various countries across age groups and surgery types [23–28] to predict postoperative complications with the goal of improved postoperative care or appropriate interventions. Unlike these studies, our goal is not to predict the postoperative outcome but to understand the association between SVI and observed outcomes. Our study differs from all the above work and contributes uniquely as follows: 1) EHR extracted patient data and expert-generated postoperative complications (labels) as compared to population-level administrative claims data and self-reported labels, 2) Multiple surgeries and multiple adverse outcomes, making the study generalizable, and 3) Using the whole population spanning across SVI values and avoiding skewed analysis. The demographic distribution of St. Louis bistate area, known for significant SVI disparities [29] and residential segregation in SDOH [30] serves as an excellent data source for such analysis supporting our goal of performing a comprehensive analysis studying the association between social vulnerability index and postoperative outcomes. We hope such analysis would be useful in designing interventions both at clinical and policy levels.

## Methods

### Patient data collection

In this study, we used data that was retrospectively extracted from the EHR and administrative records of Barnes Jewish Hospital between 2012 and 2018. The Human Research Protection Office at Washington University in St Louis, USA approved this study and granted a waiver of informed consent. The dataset contains patients' demographics, billing addresses, preoperative laboratory measurements, medical and surgical history, physical examination, surgery details, and postoperative outcomes. It is described in detail, including preprocessing steps, in [31, 32] and was used in several previous studies [33–38]. We included only addresses in Missouri and Illinois, as the density of patients outside these states was too low. We included only patients who were above 18 years of age. We removed patient cases with missing data for any of the variables included in the model. Thus as the model level increased, the number of patients decreased due to the compounding effect of missing data. The resulting number of patients without removing data for missing outcomes was 57,811. Different outcomes had different patterns of missingness, which are summarized in Table S1. All outcomes but delirium had over 50,000 cases.

We retrieved SVI values from the Center for Disease Control (CDC) released for 2018 [12]. We mapped patient addresses to census tracts using ArcGIS Pro with the Business Analyst toolbox and aggregated outcome rates by census tract for visualization. We mapped the SVI data by census tract to each patient. We also mapped the patients to their area deprivation index [39] values for 2015 using ZIP + 4 codes.

We selected a group of postoperative outcomes to analyze: 30-day mortality, congestive heart failure, leg blood clot, heart attack, surgical wound infection, pneumonia, nerve injury, abnormal heart rhythm, acute kidney injury, and delirium. All outcomes are incident, and they are defined in prior work [31]. We selected all possible physician confirmed outcomes and only excluded complications with very low incidence (less than 0.75%) as those would yield unreliable statistical results. Out of all the outcomes, delirium had a lower number of tested cases and a high incidence rate (Table 1) as it was evaluated using the confusion assessment method [40] which was only conducted on a subset of patients.

**Table 1:** Participant cohort statistical characteristics. Confusion Assessment Method (manually expensive) was performed only on a subset of patients identified and hence higher incidence rate for delirium

Characteristic	Total (*n* = 57,811)	Male (*n* = 27,980)	Female (*n* = 29,831)
Age, Median (IQR)	57 (23)	58 (22)	55 (24)
Sex, n (%)	57,819	27,980 (48.40%)	29,831 (51.60%)
BMI, Median (IQR)	28.65 (9.3)	28.32 (7.9)	29.04 (10.8)
ASA Physical Status, Median (IQR)	2.0 (1.0)	3.0 (1.0)	2.0 (1.0)
Functional Capacity, Median (IQR) 1.0 = > 10 METS 2.0 = 6–10 METS 3.0 = 4–6 METS 4.0 = < 4 METS	2.0 (1.0)	2.0 (1.0)	2.0 (1.0)
Smoking Status Yes N (%)	27,263 (52.38%)	14,742 (59.13%)	12,521 (46.16%)
Diabetes N (%)	4139 (7.95%)	2120 (8.50%)	2019 (7.44%)
Surgery Type N (%)			
Orthopedic	14,521 (25.1)	7087 (25.3)	7434 (24.9)
Gynecology	6495 (11.2)	7 (0.03)	6488 (21.7)
Cardiothoracic	6227 (10.8)	1053 (12.9)	2614 (8.8)
Urology	5903 (10.2)	4062 (14.5)	1841 (6.2)
Neurosurgery	5250 (9.1)	2773 (9.9)	2477 (8.3)
Otolaryngology	4236 (7.3)	2441 (8.7)	1795 (6.0)
General Surgery	3643 (6.3)	1983 (7.1)	1660 (5.6)
Vascular	3145 (5.4)	1677 (6.0)	1468 (4.9)
Colorectal	1994 (3.4)	1053 (3.8)	941 (3.2)
Transplant	1767 (3.1)	960 (3.4)	807 (2.7)
Hepatobiliary	1411 (2.4)	653 (2.3)	758 (2.5)
Minimally Invasive Surgery	925 (1.6)	366 (1.3)	559 (1.9)
Plastic	889 (1.5)	579 (2.1)	310 (1.0)
Unknown	805 (1.4)	369 (1.3)	436 (1.5)
Others	600 (1.0)	357 (1.3)	243 (0.8)
Race/Ethnicity N (%)			
White	43,177 (74.7)	21,302 (76.1)	21,875 (73.3)
Black	11,418 (19.8)	4963 (17.7)	6455 (21.6)
Asian	417 (0.7)	171 (0.6)	246 (0.8)
Other	55 (0.1)	22 (0.1)	33 (0.1)
Unknown	2744 (4.7)	1522 (5.4)	1222 (4.1)
Comorbidities			
Aortic Stenosis N (%)	590 (1.12)	316 (1.27)	268 (0.99)
Hypertension N (%)	11,034 (20.98)	5414 (21.72)	5522 (20.36)
Dialysis N (%)	654 (1.24)	362 (1.45)	283 (1.04)
Coronary Arterial Disease N (%)	3151 (5.99)	2081 (8.35)	1.36 (3.82)
History of Myocardial Infarction N (%)	1549 (2.95)	1036 (4.16)	494 (1.82)
Congestive Heart Failure N (%)	1731 (3.29)	1028 (4.12)	683 (2.52)
Atrial Fibrillation N (%)	1044 (1.99)	643 (2.59)	390 (1.44)
Permanent Pacemaker N (%)	818 (1.56)	529 (2.12)	282 (1.04)
History of Stroke or Transient Ischemic Attack N (%)	519 (0.99)	248 (0.99)	268 (0.99)
Deep Venous Thrombosis N (%)	1376 (2.62)	654 (2.62)	710 (2.62)
Asthma N (%)	2252 (4.28)	671 (2.69)	1555 (5.73)
Pulmonary Embolism N (%)	529 (1.01)	236 (0.95)	285 (1.05)
Chronic Obstructive Pulmonary Disease N (%)	1848 (3.51)	949 (3.81)	878 (3.24)
Pulmonary Hypertension N (%)	1022 (1.94)	514 (2.06)	494 (1.82)
Obstructive Sleep Apnea N (%)	2903 (5.52)	1748 (7.01)	1127 (4.16)
Cirrhosis N (%)	212 (0.40)	119 (0.48)	90 (0.33)
Dementia N (%)	145 (0.28)	79 (0.32)	65 (0.24)
SVI-total, Median (IQR)	0.48 (0.49)	0.48 (0.49)	0.49 (0.50)
SVI-Socioeconomic Status, Median (IQR)	0.49 (0.51)	0.47 (0.50)	0.50 (0.51)
SVI-Household Characteristics, Median (IQR)	0.55 (0.50)	0.54 (0.50)	0.55 (0.50)
SVI-Racial and Ethnic Minority Status, Median (IQR)	0.40 (0.47)	0.38 (0.48)	0.41 (0.48)
SVI-Housing Type/Transportation, Median (IQR)	0.52 (0.48)	0.51 (0.49)	0.53 (0.48)
Outcomes N (%)			
30-day Mortality	514 (0.978)	280 (1.123)	229 (0.844)
Congestive Heart Failure	709 (1.348)	431 (1.729)	269 (0.992)
Leg Blood Clot	536 (1.019)	305 (1.223)	224 (0.826)
Heart Attack	396 (0.753)	247 (0.991)	140 (0.516)
Surgical Wound Infection	623 (1.185)	339 (1.36)	273 (1.007)
Pneumonia	830 (1.578)	460 (1.845)	365 (1.346)
Nerve Injury	827 (1.573)	423 (1.697)	393 (1.449)
Abnormal Heart Rhythm	2483 (4.722)	1501 (6.021)	955 (3.521)
Acute Kidney Injury	2650 (5.242)	1567 (6.565)	1066 (4.054)
Delirium	1695 (40.992)	967 (39.909)	716 (42.267)

### Statistical analyses

We constructed three levels of modeling to account for different covariate factors in each level. For each outcome, we fit binary logistic regression models of the postoperative outcomes with the overall summary percentile ranking of SVI (RPL_THEMES) as an independent variable. We chose this linear modeling scheme as it reduces the chances of overfitting and can also estimate the gradual effects of social vulnerability variables on the incidence of postoperative complications. It also allows us to test specific interaction terms as done in subsequent analyses. We controlled for age, sex, race, and surgical speciality for level 0 and additionally for body mass index (BMI), smoking status, and diabetes for the level 1 model. In level 2 modeling, we added preoperative ASA physical status, functional capacity and comorbidities (aortic stenosis, hypertension, dialysis, coronary arterial disease, history of myocardial infarction, congestive heart failure, atrial fibrillation, history of stroke or transient ischemic attack, deep venous thrombosis, asthma, pulmonary embolism, chronic obstructive pulmonary disease, pulmonary hypertension, obstructive sleep apnea, cirrhosis, and dementia). We selected these three levels of modeling where the zeroth level included basic demographic factors, first level included preoperative variables that do not necessarily relate to the relationship between SVI and postoperative complications, and the second level including basic health and comorbidities that are likely to be related to the SVI-postoperative complications relationship. From these model coefficients, the odds ratio is reported between the two ends of ranking of the SVI values. Models with an SVI:sex and SVI:race interaction term were also computed. Additionally, we also computed models for the four themes of SVI using the level 1 implementation. For the main and interaction effects, we accounted for multiple comparisons using the Benjamini–Hochberg false discovery rate correction [41]. For the fine grained theme-based effect models, we did not apply multiple comparison correction as associations were already confirmed from the main effects models.

The models and analyses were all conducted in Python (v3.6.7) using the statsmodels toolbox (v0.12.2). For data handling, we used the pandas library (v1.1.5) and for plotting we used the matplotlib (v3.3.4) and seaborn (v0.11.2) libraries.

## Results

**Fig. 1:**
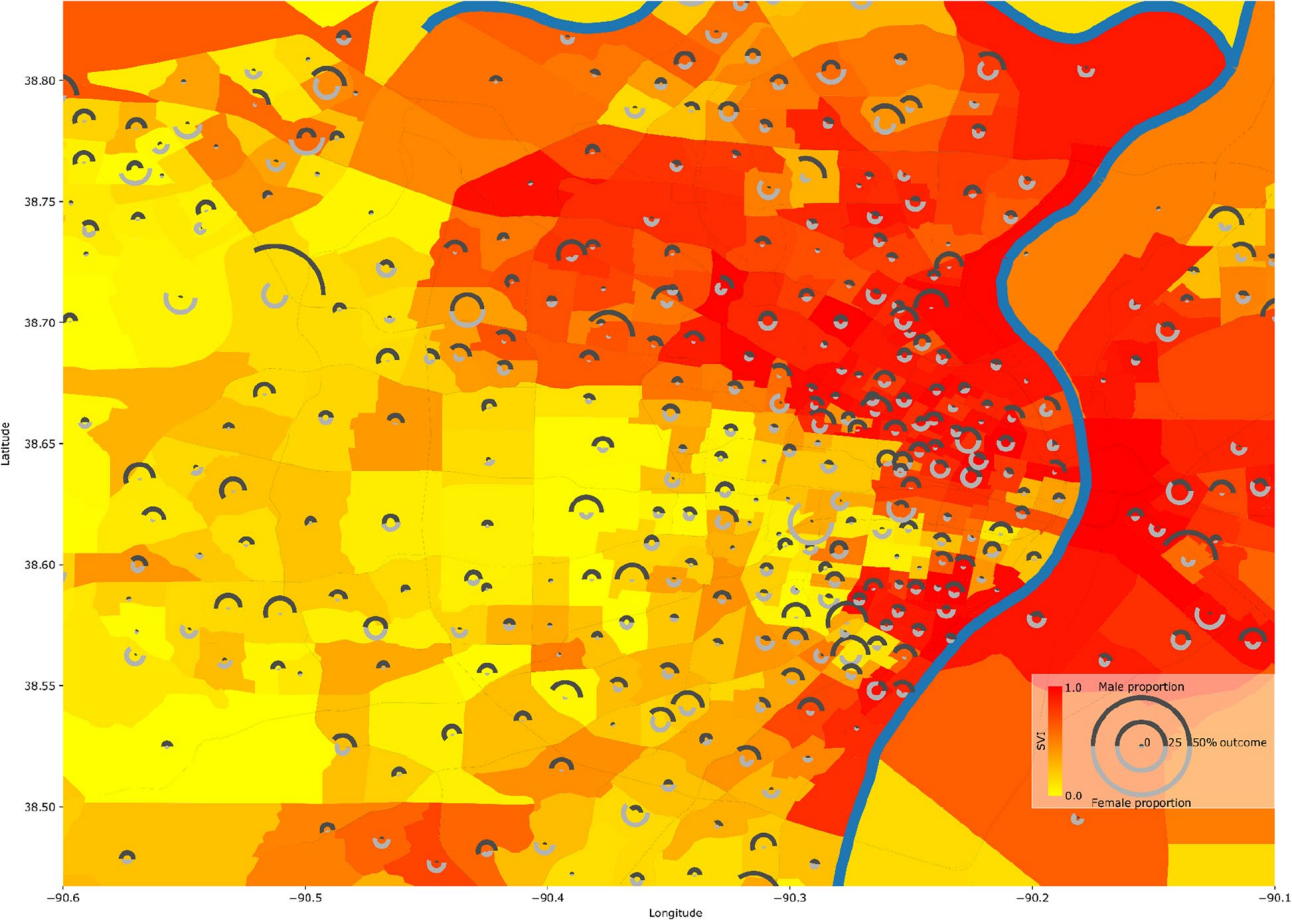
Sample outcome mapped with social vulnerability index (SVI): Mapping of SVI in counties of St. Louis bistate area with the odds of a sample outcome (abnormal heart rhythm) stratified by sex-at-birth for each census tract. Background color specifies the SVI value for the census tract and the pie chart radius indicates the proportion of the census tract population with the adverse outcome while the portion of the pie indicates the sex percentages. Counties with less than 20 subjects are omitted

**Fig. 2:**
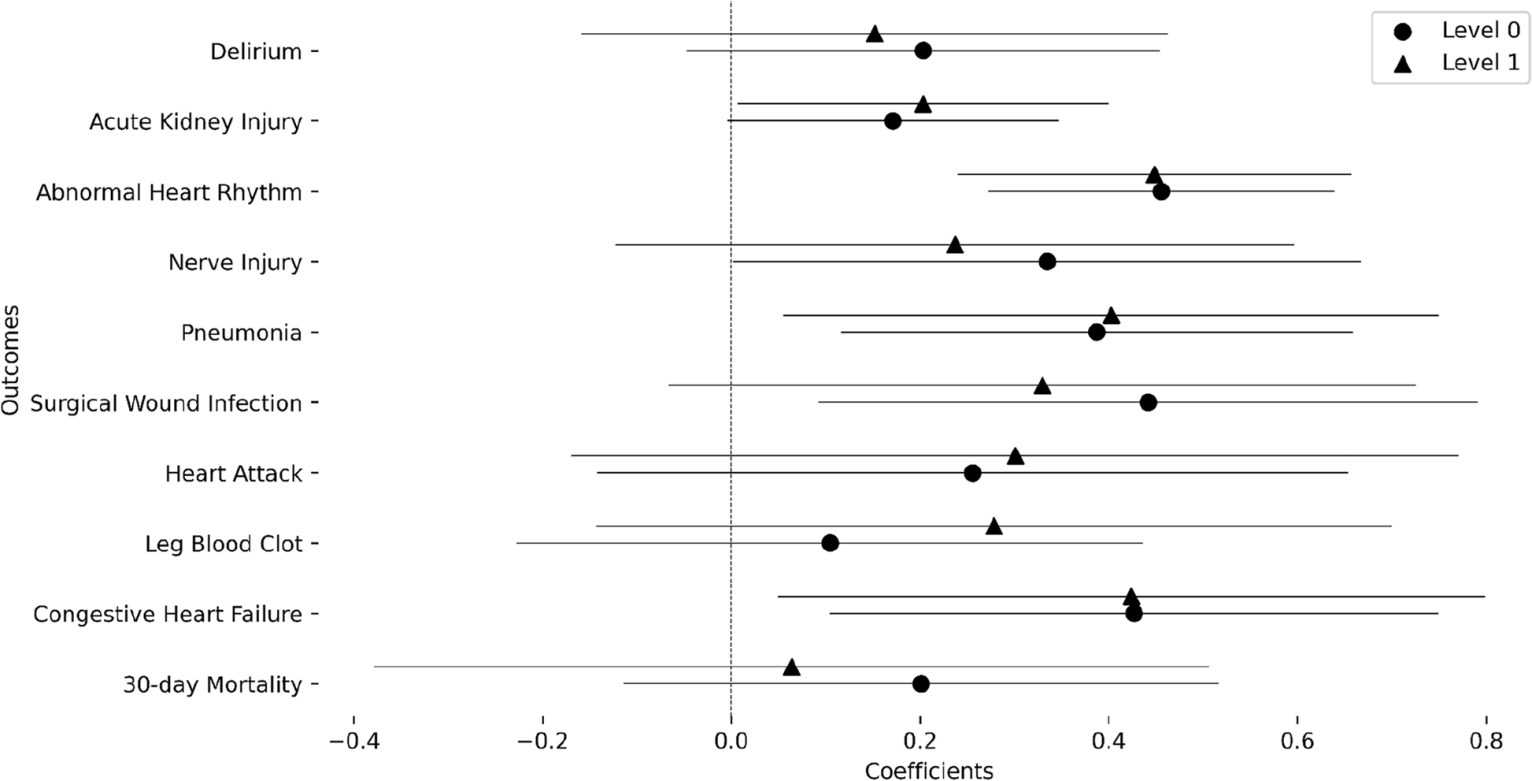
Coefficients of the overall SVI value in the association models for each outcome: Results of each level are plotted with uncorrected 95% confidence intervals

Table 1 contains descriptive statistics of the included population. Female participants had a higher SVI albeit with a small difference in medians (Median SVI: Male = 0.48, Female = 0.49). Figure 1 shows a map of the St. Louis bistate area overlaid with a representative result of the abnormal heart rhythm outcome rate stratified per census tract and sex with sex percentages indicated. It also depicts the social vulnerability index (SVI) that shows a high discrepancy in SVI where the areas in the north and around the Mississippi river show relatively high SVI in comparison to the south western regions. The transition between low and high SVIs is abrupt and indicates the high levels of inequality within the community. It also visually depicts the increase in abnormal heart rhythm frequency in areas of high social vulnerability. This is further depicted in Fig. 2 where the coefficient of the SVI is 0.456 (*p*_FDR_ = 1.14 × 10^–5^) indicating that a transition from an SVI of 0 to 1 is associated with an increase in the odds of abnormal heart rhythm by 1.577 times. In addition to observing the above mentioned significant association at the level 0 modeling variables containing age, sex, race, and surgery speciality, this significant relation was also observed in level 1 of modeling that includes smoking, diabetes status, and body mass index (coefficient = 0.448, *p*_FDR_ = 2.54 × 10^–4^, odds increase = 1.564). However, for congestive heart failure (coefficient = 0.427, *p*_FDR_ = 0.031, odds increase = 1.532), surgical wound infection (coefficient = 0.442, *p*_FDR_ = 0.033, odds increase = 1.555), and pneumonia (coefficient = 0.387, *p*_FDR_ = 0.026, odds increase = 1.473) the significant association of increasing outcome odds with an increase in SVI was only observed at level zero modeling but not at level one. The remaining outcomes did not show significant associations at any level. Level 2 modeling did not yield any significant difference (Figure S1) which suggests that preoperative health and existence of comorbidities affects the relationship between SVI and postoperative complications. These results were also corroborated by the results investigating the effect using area deprivation index (ADI) that showed consistent results with additional significant results for pneumonia and acute kidney injury outcomes (Figure S2 and Table S2). Complete statistical results are shown in Table 2.

**Table 2:** Association of SVI with postoperative outcomes. It shows odds ratio and *p*-values. Bolded rows are statistically significant after false discovery rate correction

Outcome Variable	Level	Odds ratio (CI)	*p*-value	*Adjusted p-*value
30-day Mortality	0	1.223 (1.370)	0.211	0.234
	1	1.066 (1.557)	0.777	0.777
	2	0.547 (2.045)	0.099	0.944
Congestive Heart Failure	**0**	**1.532 (1.380)**	**9.45 × 10**^**–3**^	**0.031**
	1	1.527 (1.454)	0.027	0.089
	2	1.021 (1.781)	0.944	0.944
Leg Blood Clot	0	1.110 (1.393)	0.537	0.537
	1	1.321 (1.526)	0.196	0.263
	2	1.172 (1.975)	0.647	0.944
Heart Attack	0	1.292 (1.489)	0.207	0.234
	1	1.351 (1.599)	0.210	0.263
	2	0.760 (2.042)	0.451	0.944
Surgical Wound Infection	**0**	**1.555 (1.416)**	**0.013**	**0.033**
	1	1.391 (1.486)	0.103	0.205
	2	0.869 (1.842)	0.653	0.944
Pneumonia	**0**	**1.473 (1.312)**	**0.005**	**0.026**
	1	1.497 (1.414)	0.023	0.089
	2	1.092 (1.738)	0.756	0.944
Nerve Injury	0	1.398 (1.396)	0.049	0.094
	1	1.267 (1.432)	0.196	0.263
	2	1.418 (1.779)	0.234	0.944
**Abnormal Heart Rhythm**	**0**	**1.577 (1.202)**	**1.14 × 10**^**–6**^	**1.14 × 10**^**–5**^
	**1**	**1.564 (1.234)**	**2.54 × 10**^**–5**^	**2.54 × 10**^**–4**^
	2	1.064 (1.391)	0.712	0.944
Acute Kidney Injury	0	1.186 (1.193)	0.056	0.094
	1	1.225 (1.218)	0.043	0.107
	2	1.066 (1.376)	0.695	0.944
Delirium	0	1.225 (1.285)	0.112	0.160
	1	1.164 (1.364)	0.338	0.375
	2	1.018 (1.633)	0.941	0.944

**Fig. 3:**
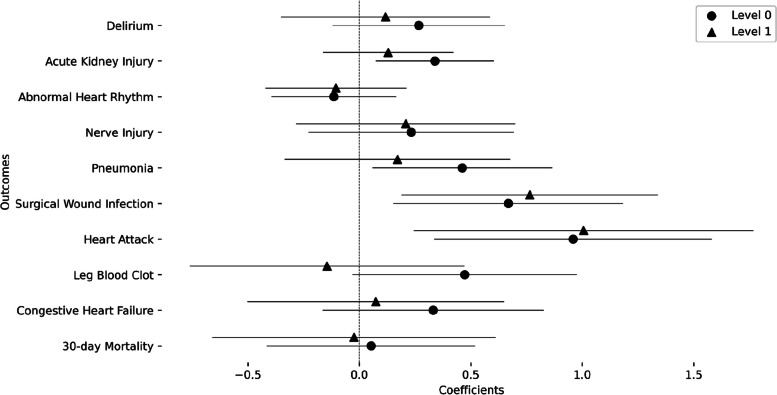
Coefficients of the interaction of overall SVI value with sex in the association models for each outcome: Results of each level are plotted with uncorrected 95% confidence intervals

In order to investigate the effect on different sexes-at-birth, we added a sex interaction term (Fig. 3). It showed that SVI associations were further increased by sex being female for the surgical wound infection (coefficient = 0.668, *p*_FDR_ = 0.034, odds increase = 1.950) and heart attack (coefficient = 0.959, *p*_FDR_ = 0.025, odds increase = 2.610) outcomes. This was robust for level 1 as well in both outcomes (Surgical wound infection: coefficient = 0.764,* p* = 0.048, odds increase = 2.145; Heart attack: coefficient = 1.005, *p*_FDR_ = 0.048, odds increase = 2.734). While males had an overall higher possibility of developing these adverse outcomes, females were more susceptible to an increase in probability of developing these outcomes in association with SVI (Figure S3). Surgical wound infection also showed significant interaction with ADI (Figure S4 and Table S3). The remaining outcomes did not show any significant sex interaction, and level 2 modeling did not show any significant result (Figure S5). Complete statistical results are shown in Table 3. We also investigated the race interaction with SVI but it did not yield any significant results (Figure S6 and Table S4).

**Table 3:** Interaction term of SVI and female sex for post-surgical complications. Bolded rows are statistically significant after false discovery rate correction

Outcome Variable	Level	Odds ratio (CI)	*p*-value	*Adjusted p-*value
30-day Mortality	0	1.054 (1.373)	0.824	0.824
	1	0.976 (1.457)	0.941	0.941
	2	0.658 (2.848)	0.435	0.985
Congestive Heart Failure	0	1.504 (1.399)	0.191	0.273
	1	1.076 (1.739)	0.803	0.892
	2	0.978 (1.742)	0.942	0.985
Leg Blood Clot	0	1.601 (1.469)	0.066	0.132
	1	0.865 (1.525)	0.644	0.805
	2	1.010 (1.020)	0.985	0.985
**Heart Attack**	**0**	**2.610 (1.314)**	**0.003**	**0.025**
	**1**	**2.734 (1.080)**	**0.010**	**0.048**
	2	1.981 (1.620)	0.246	0.985
**Surgical Wound Infection**	**0**	**1.950 (1.413)**	**0.011**	**0.034**
	**1**	**2.145 (1.486)**	**0.009**	**0.048**
	2	1.415 (1.321)	0.025	0.062
Pneumonia	0	1.187 (1.506)	0.507	0.805
	1	0.770 (2.220)	0.513	0.985
	2	1.262 (1.385)	0.322	0.402
Nerve Injury	0	1.232 (1.247)	0.406	0.805
	1	1.010 (1.011)	0.985	0.985
	2	0.891 (1.479)	0.420	0.467
Abnormal Heart Rhythm	0	0.900 (1.371)	0.513	0.805
	1	0.858 (1.655)	0.544	0.985
	2	1.401 (1.329)	0.012	0.041
Acute Kidney Injury	0	1.138 (1.633)	0.387	0.805
	1	1.030 (1.030)	0.781	0.985
	2	1.303 (1.352)	0.176	0.273
Delirium	0	1.124 (1.469)	0.624	0.805
	1	1.111 (1.246)	0.781	0.985
	2	1.029 (1.100)	0.939	0.985

**Fig. 4:**
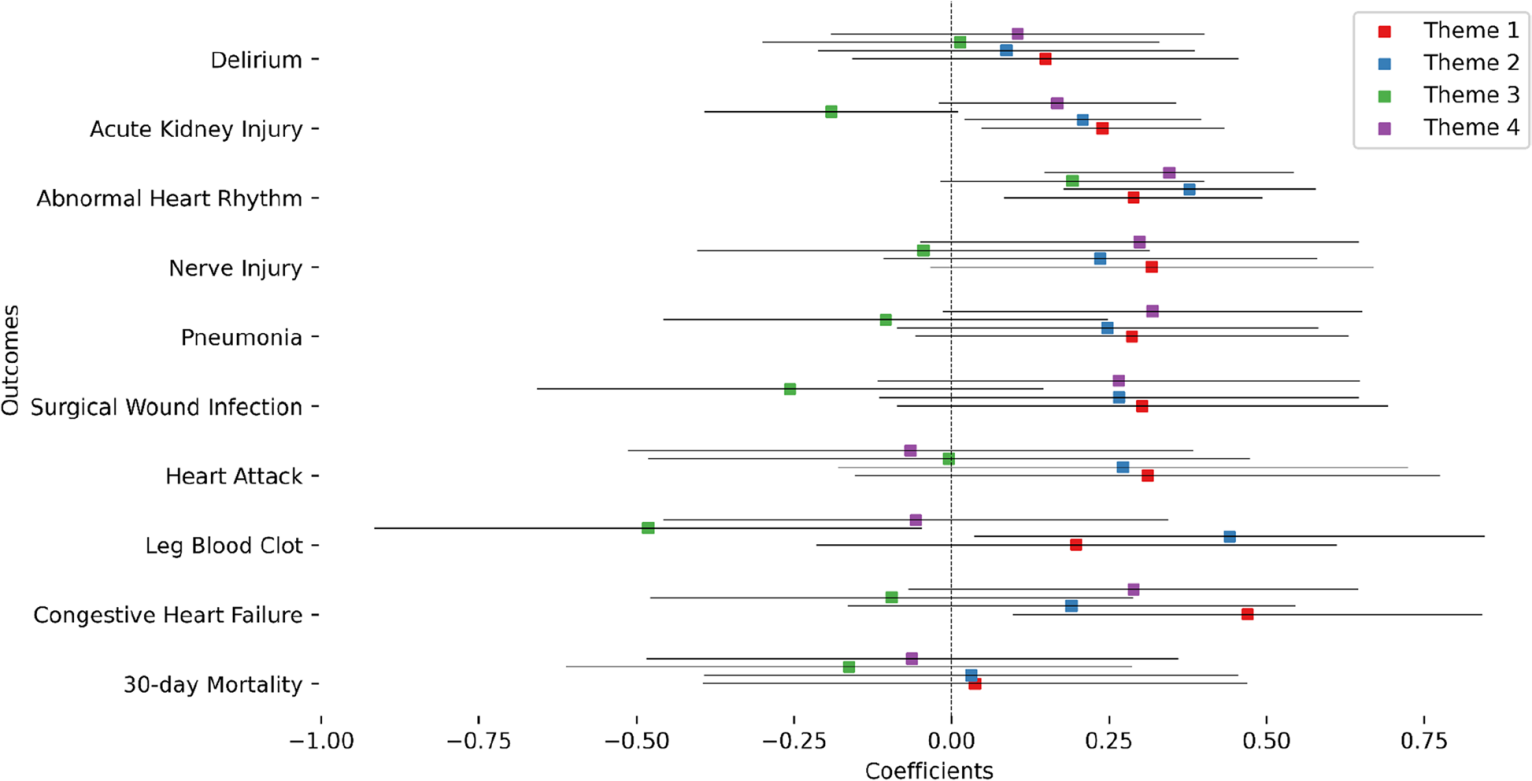
Coefficients of the theme-based SVI value in the association models for each outcome: Results of level 1 modeling for each theme are plotted with uncorrected 95% confidence intervals

**Fig. 5:**
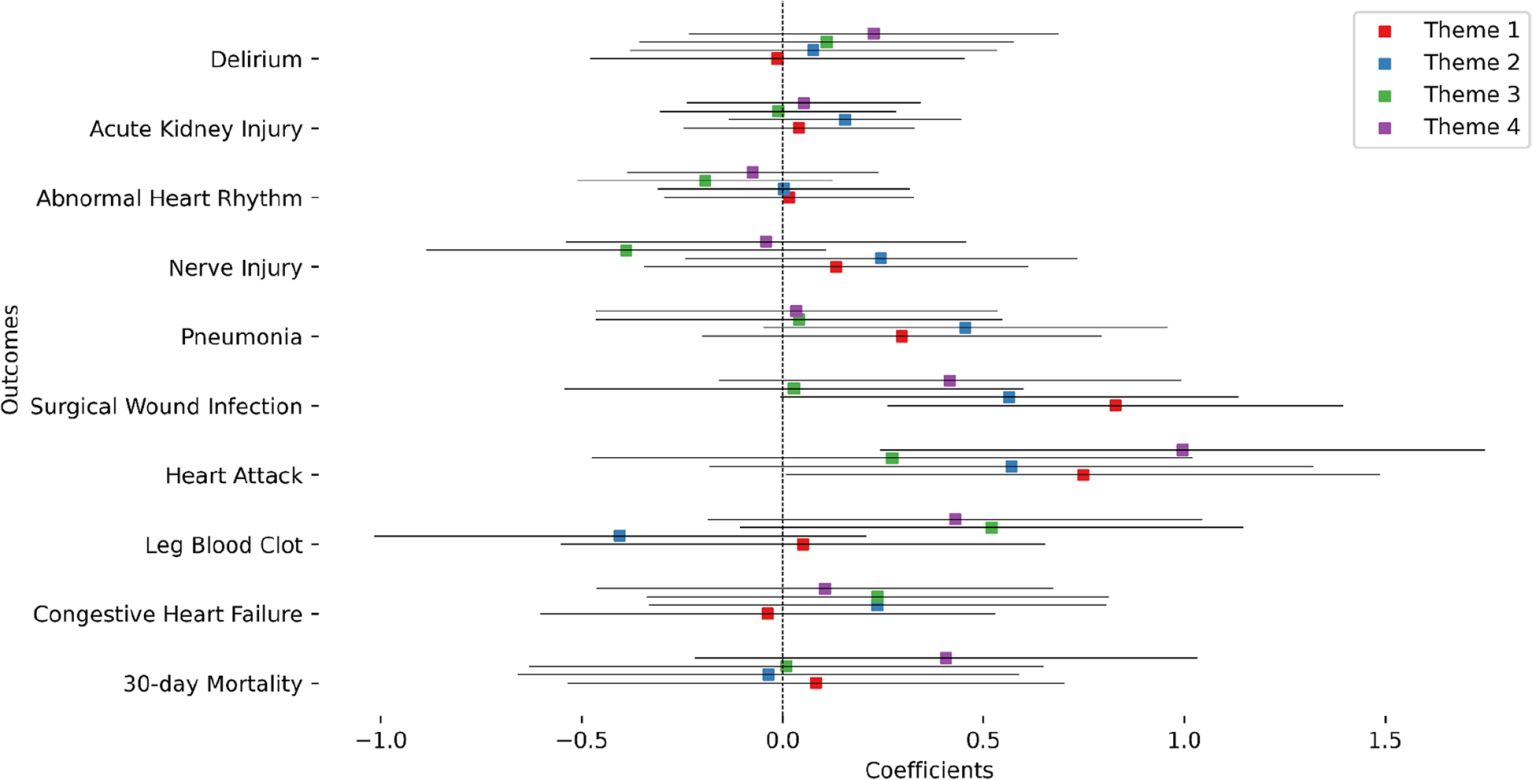
Coefficients of the interaction of theme-based SVI value with sex in the association models for each outcome: Results of level 1 modeling for each theme are plotted with uncorrected 95% confidence intervals

We then performed a more fine grained analysis of the theme-based social vulnerability index to pinpoint the form of vulnerability that contributes the most to the increase in odds of post-surgical outcomes. We first investigated the correlations between the different themes and their correlation to the overall SVI (Figure S7). Theme 1 had the highest correlation with the overall SVI while theme 3 had the lowest. Theme 2 and 3 had a negative correlation while themes 1 and 2 had the highest correlation. Theme 1 (Socioeconomic status) and theme 2 (Household composition & disability) had the most abundant significant main associations with three outcomes each, namely acute kidney injury, abnormal heart rhythm, and congestive heart failure for theme 1 and acute kidney injury, abnormal heart failure, and leg blood clot for theme 2 (Fig. 4 and Table S5). Theme 3 (Minority status and language) and theme 4 (Housing type and transportation) had one significant association each. Oddly, minority status and language had a negative coefficient meaning it contributed to a decrease in the odds of leg blood clot outcome (coefficient = -0.481, *p* = 0.030, odds = 0.617). Sex interaction analysis (Fig. 5 and Table S6) showed significant associations for theme 1 for both surgical wound infection and heart attack. Theme 4 had a significant interaction term association only for the heart attack outcome. In both cases, the significant interactions indicated an increase in odds for females.

## Discussion

In this study, we investigated the association of tract-wide social vulnerability with the outcomes of surgical operations. Our study’s unique contribution lies in investigating this association in a historically segregated area while utilizing a large number of physician confirmed postoperative complications across various surgeries.

We found abnormal heart rhythm, a cardiovascular complication, to be associated with the overall vulnerability. Socioeconomic status and housing status were some of the vulnerabilities that contributed to the overall associations with the outcome complications.

Our findings are in line with previous literature connecting social vulnerability with health outcomes where Hyer et al. [14] showed that cancer patients from high-SVI counties more frequently had a postoperative complication and had an extended length of stay. Further studies also showed the association between SVI and the overall group of post-operative complications that includes pulmonary failure, pneumonia, myocardial infarction, deep venous thrombosis, pulmonary embolism, renal failure, surgical site infection, gastrointestinal bleeding, and postoperative hemorrhage [15, 16]. Stuart et al. [18] studied individual postoperative complications and found that esophagectomy patients with high SVI had greater rates of pneumonia, jejunal feeding-tube complications, and unplanned readmission. Carmichael et al. [17] also studied specific post-operative complications and found that colorectal surgery patients in the highest SVI quartile have an increased risk of mechanical ventilation for more than 48 h postoperatively, surgical site infection, and sepsis/septic shock. We noticed that SVI had a significant impact on certain outcomes but not on others. This can be attributed to the specific causes behind the development of each outcome and the associated risk factors. For instance, risk factors for abnormal heart rhythm may include diabetes, high blood pressure, smoking, stress, anxiety, and more. These risk factors are usually exacerbated for individuals living in poverty, unemployed, elderly, or lacking stable housing. However, the risk factors for other outcomes might differ, and their connection to factors contributing to SVI may vary.

We also found other complications such as congestive heart failure, surgical wound infection, and pneumonia that have significant SVI association only at the level zero modeling. This suggests that smoking, diabetes, and unhealthy BMI alters the relationship between SVI and postoperative complications. Our results are consistent with the existing literature across surgeries for diabetes and unhealthy BMI as associated factors. Covarrubias et al. [42] observed that the likelihood of postoperative pulmonary complications increased with increasing body mass index in patients undergoing trauma laparotomy. Zhang et al. [43] found that lower body mass index and the presence of diabetes is a risk factor for postoperative pneumonia in craniotomy patients. A systematic review [44] noted that obese patients had significantly higher odds of postoperative atrial fibrillation when compared with non-obese patients undergoing cardiac surgery. Regarding smoking, our results agree with that of [45], where postoperative delirium was shown to be higher in patients with smoking history. However, our results challenge the findings of [46, 47] that smoking was not associated with medical complications (myocardial infarction, pneumonia, etc.) but is associated with surgical complications in arthroplasty patients. These all point to the fact that the effect of SVI can be observed through the general health of the patients entering surgery due to smoking and food choices.

By investigating the sex interaction terms, we found that heart attack and surgical wound infection were specifically associated with females. Previous studies noted the increased occurrence of post-surgical complications in females. Sah et al. [48] noted that females have a higher incidence of postoperative complications (as defined by POSSUM) after gastric cancer surgery and found patient gender to be a risk factor for postoperative complications. Aghdassi et al. [49] investigated gender as a risk factor for surgical site infections (SSI) and found that even though the incidence rate ratio and the adjusted odds ratio for SSI were significantly higher for male patients, for heart and vascular surgery, SSI rates were significantly higher for female patients. Kim et al. [50] identified that female sex is one of the risk factors for postoperative hematoma after biportal endoscopic spinal surgery. The reason for this increased risk is unclear but it could be possibly related to caregiving responsibilities carried out traditionally by women as these responsibilities can exacerbate poverty levels due to inability to seek employment. This theory is supported by the relationship between poverty and poor quality of life in people with caregiving burden [51]. Other possible interpretations include gender-based violence as a factor of health [52] which is known to increase in areas of lower socioeconomic status [53]. Access to reproductive and sexual health products could also be another factor as it is also known to be related to socioeconomic status, especially in the United States context [54].

We also found that out of the sub-indices for vulnerability, socioeconomic status (SES) and household composition and disability were the most influential. Those two factors, in our study, are used as a proxy for the individual vulnerability of each patient. This is in contrast with the other two themes (housing type and transportation and minority status & language) where they represent the community effect on the patient since we already include age and race as covariates in our model. These covariates are sub-measures of the two themes and they are measured at the individual level. An attempt to separate these two effects is made by Bonner et al. [55] where they studied the association between racial disparities in outcomes and the corresponding healthcare cost. Our findings line up with previous studies that found that liver transplant patients with Medicaid or Medicare (a proxy for SES) had higher postoperative mortality compared to the patients with private insurance [56]. A systematic review found that socioeconomic status and patient demographics both impact the patient-reported outcomes post orthopedic surgery [57]. Measurements of SES by median neighborhood household income (categorized into quintiles) revealed that lower SES is associated with fewer days alive and out of hospital post major elective non-cardiac surgery [58]. Ambur et al. [59] used the National Inpatient Sample (NIS) database of more than 2 million patients undergoing cholecystectomy and found that SES had a negative impact on postoperative outcomes, in addition to being on Medicaid increasing postoperative mortality. Mheaffey et al. [60] investigated a sample of 44,451 patients where the distressed communities index (DCI) was used as a proxy for SES in the American College of Surgeons-National Surgical Quality Improvement Program (ACS-NSQIP) database and risk models. They found that higher DCI was associated with an increased rate of postoperative complications and resource utilization even after ACS-NSQIP risk adjustment further demonstrating surgical outcome disparity based on community level socioeconomic factors.

Social vulnerability has also been known to affect general health which could, in turn, have an effect on surgical outcomes. The effect of socioeconomic status as reflected by belonging to a certain income region and housing was particularly affecting females’ incidence of heart attack and surgical wound infection. Previous studies investigated the intersectional effect of gender and SES on internalizing symptoms and found no interactive negative effects for female gender and low SES in a sample of predominantly lower-SES adolescents [61]. However, they did observe that females at the lowest levels of caretaker education and household income (indices of SES) were at higher risk of depressive symptoms and hence could lead to premature cardiovascular disease and suboptimal cardiovascular health [62]. Leng et al. [63] showed that increased risk of hypertension in the lowest categories of all SES indicators was most evident in women, whereas men revealed less consistent associations. Hinz et al. [64] showed that individually being a female and belonging to low SES strata was associated with poor sleep quality. All those indicators of health could contribute to the disproportionate effect that females are exposed to from social vulnerability.

Our investigation here is limited by multiple factors. First of all, SVI as a proxy will tend to understate true associations vs. measuring the patient level factors. Additionally, our study population introduces biases where the population that is able to receive a surgery is the insured or the more financially stable within their regions. Also, the regional nature of the study means there are possibly region-specific variables that could bias our results. However, we argue that location specificity is actually an advantage as it provides a balanced representation of different SVIs from one region. Our results only include a limited number of covariates, while it is possible that other terms not included in our model could explain more variance such as education level, marital status, and insurance status. Finally, the use of uncorrected statistics for theme-based analysis could lead to more type-2 errors but we opted to not correct them since the relations were already established in the overall SVI models. Regardless of the biases and limitations, the results are useful for planning interventions to target preoperative optimization and postoperative support services.

These results shed light on the healthcare cost of socioeconomic disparity in the St. Louis region. They show that social vulnerability can have effects that propagate downstream into seemingly unrelated domains such as surgical outcomes by affecting the general health and functional capacity of patients making them more vulnerable to post-surgical complications with women disproportionately affected by some complications. It highlights the importance of alleviating socioeconomic pressure as a priority for the fields of preventive medicine and public health.

## Conclusions

Our results indicate that social vulnerability could be related to adverse outcomes of surgery and that females are more affected. Socioeconomic status and housing were the most significantly associated factors which indicates the importance of prioritizing welfare as a method of improving surgical outcomes. These results could serve as guidance for the public health policy field as social vulnerability could be viewed as adding an accruing cost to the healthcare system. We can also argue through these results that decreasing social vulnerability is an important step towards achieving prevention-based healthcare.

### Supplementary Information

Supplementary Material 1.

## Data Availability

The datasets generated and/or analysed during the current study are not publicly available due to containing sensitive patients’ information but are available from the corresponding authors on reasonable request.
