# Supplementary material for: Social vulnerability and surgery outcomes: a cross-sectional analysis
Source: BMC Public Health. 2024 Jul 16;24:1907. doi: 10.1186/s12889-024-19418-5 (PMC11253435; doi:10.1186/s12889-024-19418-5)
Supplement: Supplementary file 1 — Supplementary Material 1. [file 12889_2024_19418_MOESM1_ESM.docx]

Supplementary Information:

Social Vulnerability and Surgery Outcomes: A Cross-sectional Analysis

Mohamed Abdelhack^1,3✝*^; Sandhya Tripathi^1✝*^; Yixin Chen^2^; Michael S Avidan^1^; Christopher R King^1^

^1^ Department of Anesthesiology, Washington University School of Medicine, St. Louis MO

^2^ Department of Computer Science, Washington University in St. Louis, St. Louis MO

^3^ Krembil Centre for Neuroinformatics, Centre for Addiction and Mental Health, Toronto, ON

^✝^ These authors contributed equally

^*^ Correspondence should be sent to [mohamed.abdelhack.37a@kyoto-u.jp](mailto:mohamed.abdelhack.37a@kyoto-u.jp); [sandhyat@wustl.edu](mailto:sandhyat@wustl.edu), and [christopherking@wustl.edu](mailto:christopherking@wustl.edu)

Table S1: Number of samples for each outcome at each level of modeling

| **Outcome** | **Level 0** | **Level 1** | **Level 2** |
| --- | --- | --- | --- |
| 30-day Mortality | 51997 | 52582 | 21647 |
| Congestive Heart Failure | 51997 | 52582 | 21647 |
| Leg Blood Clot | 51997 | 52582 | 21647 |
| Heart Attack | 51997 | 52582 | 21647 |
| Surgical Wound Infection | 51997 | 52582 | 21647 |
| Pneumonia | 51997 | 52582 | 21647 |
| Nerve Injury | 51997 | 52582 | 21647 |
| Abnormal Heart Rhythm | 51997 | 52582 | 21647 |
| Acute Kidney Injury | 50113 | 50552 | 20834 |
| Delirium | 4102 | 4135 | 1818 |

Table S2: Association of ADI with postoperative outcomes. It shows odds ratio and p-values. Bolded rows are statistically significant after false discovery rate correction.

| **Outcome Variable** | **Level** | **Odds ratio (CI)** | ***p*-value** | ***Adjusted p-*value** |
| --- | --- | --- | --- | --- |
| 30-day Mortality | **0** | **1.005 (1.004)** | **0.015** | **0.021** |
|  | 1 | 0.998 (1.005) | 0.543 | 0.543 |
|  | 2 | 0.993 (1.008) | 0.104 | 0.715 |
| Congestive Heart Failure | **0** | **1.006 (1.004)** | **0.003** | **0.007** |
|  | 1 | 1.005 (1.004) | 0.026 | 0.051 |
|  | 2 | 1.004 (1.007) | 0.321 | 0.776 |
| Leg Blood Clot | 0 | 1.002 (1.004) | 0.308 | 0.342 |
|  | 1 | 1.002 (1.005) | 0.370 | 0.439 |
|  | 2 | 1.003 (1.008) | 0.444 | 0.776 |
| Heart Attack | 0 | 1.001 (1.005) | 0.573 | 0.573 |
|  | 1 | 1.002 (1.006) | 0.388 | 0.439 |
|  | 2 | 0.997 (1.008) | 0.415 | 0.776 |
| Surgical Wound Infection | **0** | **1.005 (1.004)** | **0.013** | **0.021** |
|  | 1 | 1.005 (1.005) | 0.024 | 0.051 |
|  | 2 | 0.999 (1.007) | 0.867 | 0.918 |
| Pneumonia | **0** | **1.006 (1.003)** | **1.30×10^-4^** | **4.32×10^-4^** |
|  | **1** | **1.005 (1.004)** | **0.014** | **0.048** |
|  | 2 | 1.002 (1.007) | 0.588 | 0.839 |
| Nerve Injury | 0 | 1.003 (1.004) | 0.167 | 0.209 |
|  | 1 | 1.002 (1.004) | 0.395 | 0.439 |
|  | 2 | 0.999 (1.007) | 0.790 | 0.918 |
| **Abnormal Heart Rhythm** | **0** | **1.004 (1.002)** | **8.2×10^-5^** | **4.12×10^-3^** |
|  | **1** | **1.004 (1.002)** | **0.002** | **0.010** |
|  | 2 | 1.001 (1.004) | 0.466 | 0.776 |
| Acute Kidney Injury | **0** | **1.005 (1.002)** | **1.8×10^-5^** | **1.85×10^-3^** |
|  | 1 | **1.004 (1.002)** | **2.51×10^-4^** | **2.51×10^-2^** |
|  | 2 | 1.003 (1.004) | 0.143 | 0.715 |
| Delirium | **0** | **1.004 (1.003)** | **0.011** | **0.021** |
|  | 1 | 1.002 (1.004) | 0.380 | 0.439 |
|  | 2 | 1.000 (1.006) | 0.918 | 0.918 |

Table S3: Interaction term of ADI and female sex for post-surgical complications. Bolded rows are statistically significant after false discovery rate correction.

| **Outcome Variable** | **Level** | **Odds ratio (CI)** | ***p*-value** | ***Adjusted p-*value** |
| --- | --- | --- | --- | --- |
| 30-day Mortality | 0 | 0.000 (0.006) | 0.935 | 0.935 |
|  | 1 | 0.003 (0.007) | 0.382 | 0.543 |
|  | 2 | -0.001 (0.012) | 0.825 | 0.989 |
| Congestive Heart Failure | 0 | 0.003 (0.006) | 0.298 | 0.422 |
|  | 1 | -0.000 (0.007) | 0.903 | 0.989 |
|  | 2 | -0.001 (0.011) | 0.842 | 0.989 |
| Leg Blood Clot | 0 | 0.004 (0.006) | 0.224 | 0.374 |
|  | 1 | 0.002 (0.007) | 0.677 | 0.966 |
|  | 2 | -0.000 (0.012) | 0.989 | 0.989 |
| Heart Attack | 0 | 0.008 (0.008) | 0.032 | 0.110 |
|  | 1 | 0.003 (0.009) | 0.467 | 0.778 |
|  | 2 | -0.001 (0.013) | 0.850 | 0.989 |
| Surgical Wound Infection | **0** | **0.010 (0.006)** | **0.002** | **0.025** |
|  | 1 | 0.009 (0.007) | 0.015 | 0.151 |
|  | 2 | 0.013 (0.011) | 0.021 | 0.213 |
| Pneumonia | 0 | 0.004 (0.005) | 0.125 | 0.250 |
|  | 1 | 0.003 (0.006) | 0.370 | 0.778 |
|  | 2 | -0.002 (0.009) | 0.605 | 0.989 |
| Nerve Injury | 0 | 0.004 (0.005) | 0.112 | 0.250 |
|  | 1 | 0.004 (0.006) | 0.189 | 0.778 |
|  | 2 | 0.003 (0.009) | 0.442 | 0.989 |
| Abnormal Heart Rhythm | 0 | -0.001 (0.003) | 0.737 | 0.819 |
|  | 1 | 0.000 (0.004) | 0.829 | 0.989 |
|  | 2 | -0.001 (0.006) | 0.646 | 0.989 |
| Acute Kidney Injury | 0 | 0.003 (0.003) | 0.033 | 0.110 |
|  | 1 | 0.002 (0.004) | 0.389 | 0.778 |
|  | 2 | 0.002 (0.006) | 0.577 | 0.989 |
| Delirium | 0 | 0.001 (0.005) | 0.668 | 0.819 |
|  | 1 | -0.000 (0.006) | 0.989 | 0.989 |
|  | 2 | 0.000 (0.009) | 0.987 | 0.989 |

Table S4: Interaction term of SVI and race for post-surgical complications with white race as the baseline. Bolded rows are statistically significant after false discovery rate correction.

| **Outcome**  **Variable** | **Race** | **Odds (CI)** | ***p*-value** | ***Adjusted p*-value** |
| --- | --- | --- | --- | --- |
| 30-day Mortality | Asian | -0.284 (4.964) | 0.911 | 0.988 |
|  | Black | -1.269 (0.892) | 0.005 | 0.053 |
|  | Unknown | -0.033 (1.172) | 0.955 | 0.955 |
|  | Other | -0.208 (12.238) | 0.973 | 0.998 |
| Congestive  Heart Failure | Asian | -0.069 (4.386) | 0.907 | 0.988 |
|  | Black | -0.243 (0.823) | 0.563 | 0.625 |
|  | Unknown | 0.035 (1.011) | 0.946 | 0.955 |
|  | Other | -0.205 (7.986) | 0.960 | 0.998 |
| Leg Blood Clot | Asian | -0.481 (8.023) | 0.866 | 0.988 |
|  | Black | -0.675 (0.863) | 0.112 | 0.251 |
|  | Unknown | 0.527 (1.298) | 0.426 | 0.955 |
|  | Other | -0.128 (11.262) | 0.964 | 0.998 |
| Heart Attack | Asian | -0.300 (6.957) | 0.988 | 0.988 |
|  | Black | -0.300 (1.113) | 0.018 | 0.092 |
|  | Unknown | 0.391 (1.478) | 0.757 | 0.757 |
|  | Other | -0.008 (5.163) | 0.980 | 0.998 |
| Surgical Wound  Infection | Asian | -0.203 (5.526) | 0.933 | 0.988 |
|  | Black | -0.929 (0.772) | 0.866 | 0.251 |
|  | Unknown | -0.209 (1.324) | 0.112 | 0.955 |
|  | Other | -0.142 (10.902) | 0.050 | 0.998 |
| Pneumonia | Asian | -0.315 (3.647) | 0.964 | 0.988 |
|  | Black | -0.623 (0.768) | 0.988 | 0.092 |
|  | Unknown | -0.934 (0.934) | 0.018 | 0.757 |
|  | Other | 0.165 (7.120) | 0.757 | 0.998 |
| Nerve Injury | Asian | -0.030 (3.981) | 0.980 | 0.988 |
|  | Black | 0.471 (0.736) | 0.933 | 0.757 |
|  | Unknown | 0.181 (1.459) | 0.970 | 0.480 |
|  | Other | 0.299 (6.939) | 0.652 | 0.955 |
| Abnormal Heart  Rhythm | Asian | 0.039 (2.043) | 0.692 | 0.998 |
|  | Black | 0.110 (0.480) | 0.992 | 0.988 |
|  | Unknown | 0.151 (0.749) | 0.947 | 0.988 |
|  | Other | 0.023 (4.474) | 0.471 | 0.988 |
| Acute Kidney  Injury | Asian | -0.068 (2.029) | 0.453 | 0.988 |
|  | Black | -0.146 (0.397) | 0.966 | 0.998 |
|  | Unknown | 0.303 (0.792) | 0.922 | 0.988 |
|  | Other | 0.097(4.516) | 0.105 | 0.988 |
| Delirium | Asian | 0.211 (4.247) | 0.262 | 0.998 |
|  | Black | -0.596 (0.720) | 0.991 | 0.988 |
|  | Unknown | 0.629 (1.100) | 0.911 | 0.988 |
|  | Other | -0.036 (6.153) | 0.005 | 0.053 |

**Table S5:** Statistical analysis results of the theme-based SVI value in the association models for each outcome. Bolded rows are statistically significant after FDR correction.

| **Outcome**  **Variable** | **Theme** | **Odds (CI)** | ***p*-value** |
| --- | --- | --- | --- |
| 30-day Mortality | Socioeconomic Status | 1.038 (1.540) | 0.865 |
|  | Household Composition and Disability | 1.031 (1.528) | 0.885 |
|  | Minority Status and Language | 0.849 (1.566) | 0.478 |
|  | Housing Type and Transportation | 0.940 (1.523) | 0.772 |
| Congestive  Heart Failure | **Socioeconomic Status** | **1.601 (1.452)** | **0.014** |
|  | Household Composition and Disability | 1.209 (1.428) | 0.294 |
|  | Minority Status and Language | 0.909 (1.467) | 0.627 |
|  | Housing Type and Transportation | 1.334 (1.429) | 0.113 |
| Leg Blood Clot | Socioeconomic Status | 1.219 (1.511) | 0.347 |
|  | **Household Composition and Disability** | **1.554 (1.499)** | **0.033** |
|  | **Minority Status and Language** | **0.617 (1.544)** | **0.030** |
|  | Housing Type and Transportation | 0.946 (1.493) | 0.783 |
| Heart Attack | Socioeconomic Status | 1.365 (1.590) | 0.189 |
|  | Household Composition and Disability | 1.312 (1.571) | 0.238 |
|  | Minority Status and Language | 0.996 (1.610) | 0.987 |
|  | Housing Type and Transportation | 0.937 (1.566) | 0.776 |
| Surgical Wound  Infection | Socioeconomic Status | 1.353 (1.477) | 0.128 |
|  | Household Composition and Disability | 1.305 (1.464) | 0.172 |
|  | Minority Status and Language | 0.774 (1.494) | 0.211 |
|  | Housing Type and Transportation | 1.304 (1.468) | 0.175 |
| Pneumonia | Socioeconomic Status | 1.330 (1.411) | 0.103 |
|  | Household Composition and Disability | 1.282 (1.398) | 0.147 |
|  | Minority Status and Language | 0.900 (1.424) | 0.561 |
|  | Housing Type and Transportation | 1.376 (1.396) | 0.060 |
| Nerve Injury | Socioeconomic Status | 1.374 (1.421) | 0.077 |
|  | Household Composition and Disability | 1.267 (1.412) | 0.179 |
|  | Minority Status and Language | 0.957 (1.433) | 0.809 |
|  | Housing Type and Transportation | 1.348 (1.416) | 0.092 |
| Abnormal Heart  Rhythm | **Socioeconomic Status** | **1.333 (1.229)** | **0.006** |
|  | **Household Composition and Disability** | **1.459 (1.223)** | **2.28×10^-4^** |
|  | Minority Status and Language | 1.211 (1.232) | 0.072 |
|  | **Housing Type and Transportation** | **1.414 (1.220)** | **6.40×10^-4^** |
| Acute Kidney  Injury | **Socioeconomic Status** | **1.271 (1.213)** | **0.015** |
|  | **Household Composition and Disability** | **1.231 (1.207)** | **0.030** |
|  | Minority Status and Language | 0.827 (1.223) | 0.063 |
|  | Housing Type and Transportation | 1.183 (1.207) | 0.080 |
| Delirium | Socioeconomic Status | 1.161 (1.359) | 0.339 |
|  | Household Composition and Disability | 1.091 (1.349) | 0.567 |
|  | Minority Status and Language | 1.014 (1.371) | 0.929 |
|  | Housing Type and Transportation | 1.111 (1.344) | 0.488 |

**sTable S6:** Statistical analysis results of the interaction of theme-based SVI value with sex in the association models for each outcome. Bolded rows are statistically significant.

| **Outcome**  **Variable** | **Theme** | **Odds (CI)** | ***p*-value** |
| --- | --- | --- | --- |
| 30-day  Mortality | Socioeconomic Status | 1.087 (1.858) | 0.791 |
|  | Household Composition and Disability | 0.966 (1.867) | 0.913 |
|  | Minority Status and Language | 1.010 (1.896) | 0.977 |
|  | Housing Type and Transportation | 1.503 (1.870) | 0.202 |
| Congestive  Heart Failure | Socioeconomic Status | 0.964 (1.764) | 0.898 |
|  | Household Composition and Disability | 1.267 (1.766) | 0.416 |
|  | Minority Status and Language | 1.267 (1.777) | 0.419 |
|  | Housing Type and Transportation | 1.112 (1.768) | 0.716 |
| Leg Blood Clot | Socioeconomic Status | 1.052 (1.827) | 0.869 |
|  | Household Composition and Disability | 0.667 (1.844) | 0.195 |
|  | Minority Status and Language | 1.684 (1.872) | 0.103 |
|  | Housing Type and Transportation | 1.535 (1.850) | 0.171 |
| Heart Attack | **Socioeconomic Status** | **2.112 (2.092)** | **0.047** |
|  | Household Composition and Disability | 1.769 (2.121) | 0.137 |
|  | Minority Status and Language | 1.314 (2.111) | 0.474 |
|  | **Housing Type and Transportation** | **2.704 (2.125)** | **0.010** |
| Surgical  Wound  Infection | **Socioeconomic Status** | **2.292 (1.765)** | **0.004** |
|  | Household Composition and Disability | 1.760 (1.770) | 0.052 |
|  | Minority Status and Language | 1.030 (1.772) | 0.922 |
|  | Housing Type and Transportation | 1.518 (1.781) | 0.156 |
| Pneumonia | Socioeconomic Status | 1.346 (1.645) | 0.243 |
|  | Household Composition and Disability | 1.576 (1.653) | 0.076 |
|  | Minority Status and Language | 1.042 (1.658) | 0.872 |
|  | Housing Type and Transportation | 1.036 (1.646) | 0.892 |
| Nerve Injury | Socioeconomic Status | 1.143 (1.614) | 0.586 |
|  | Household Composition and Disability | 1.278 (1.629) | 0.325 |
|  | Minority Status and Language | 0.678 (1.643) | 0.125 |
|  | Housing Type and Transportation | 0.960 (1.645) | 0.873 |
| Abnormal  Heart Rhythm | Socioeconomic Status | 1.016 (1.363) | 0.920 |
|  | Household Composition and Disability | 1.003 (1.369) | 0.983 |
|  | Minority Status and Language | 0.824 (1.373) | 0.233 |
|  | Housing Type and Transportation | 0.929 (1.367) | 0.640 |
| Acute Kidney  Injury | Socioeconomic Status | 1.042 (1.334) | 0.780 |
|  | Household Composition and Disability | 1.168 (1.336) | 0.294 |
|  | Minority Status and Language | 0.989 (1.344) | 0.942 |
|  | Housing Type and Transportation | 1.054 (1.339) | 0.724 |
| Delirium | Socioeconomic Status | 0.987 (1.593) | 0.957 |
|  | Household Composition and Disability | 1.080 (1.578) | 0.741 |
|  | Minority Status and Language | 1.116 (1.594) | 0.644 |
|  | Housing Type and Transportation | 1.255 (1.582) | 0.333 |

#

#
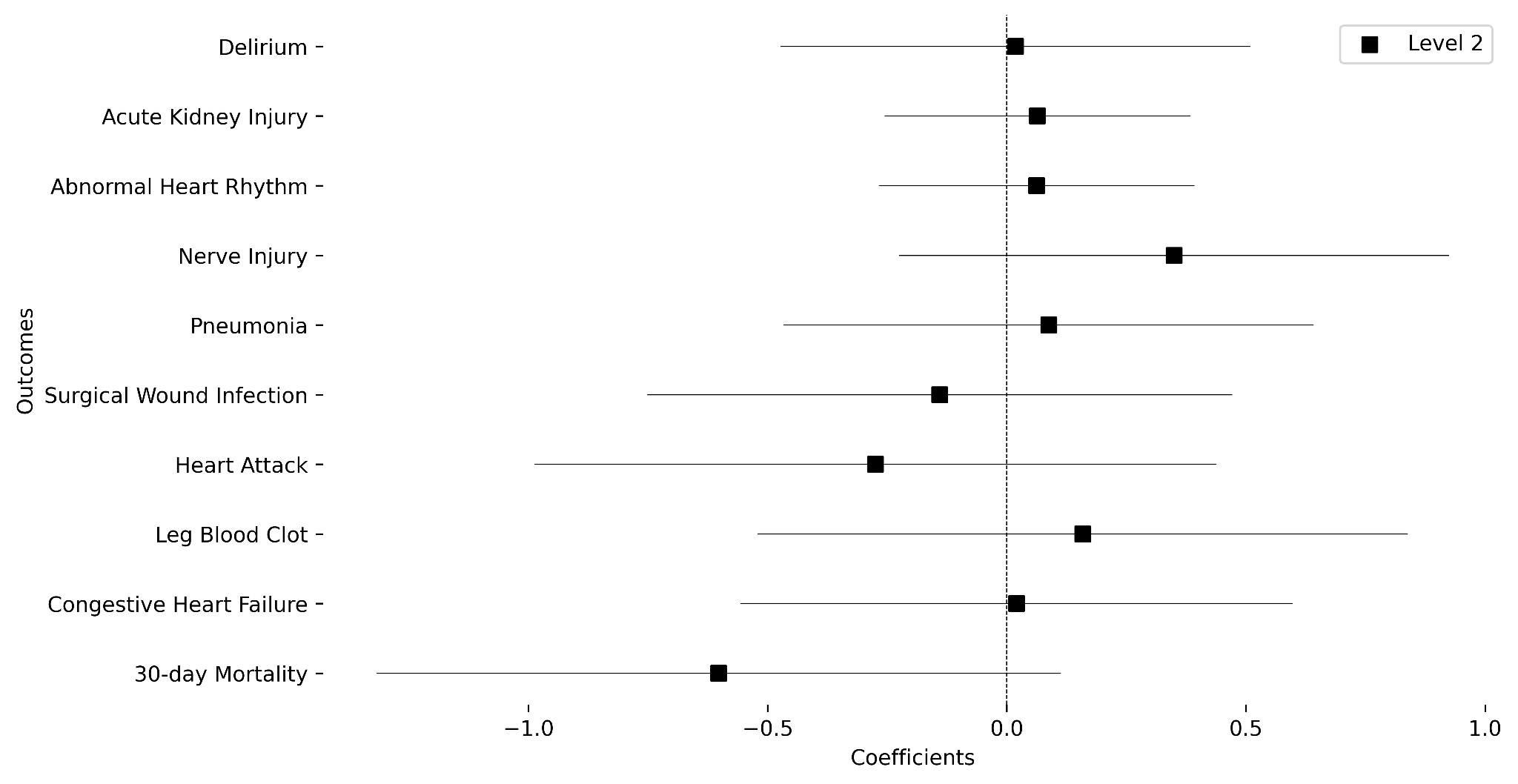


**Supplementary Figure 1: Coefficients of the overall SVI value in the association models for each outcome for level 2 models:** Results of each level are plotted with uncorrected 95% confidence intervals.


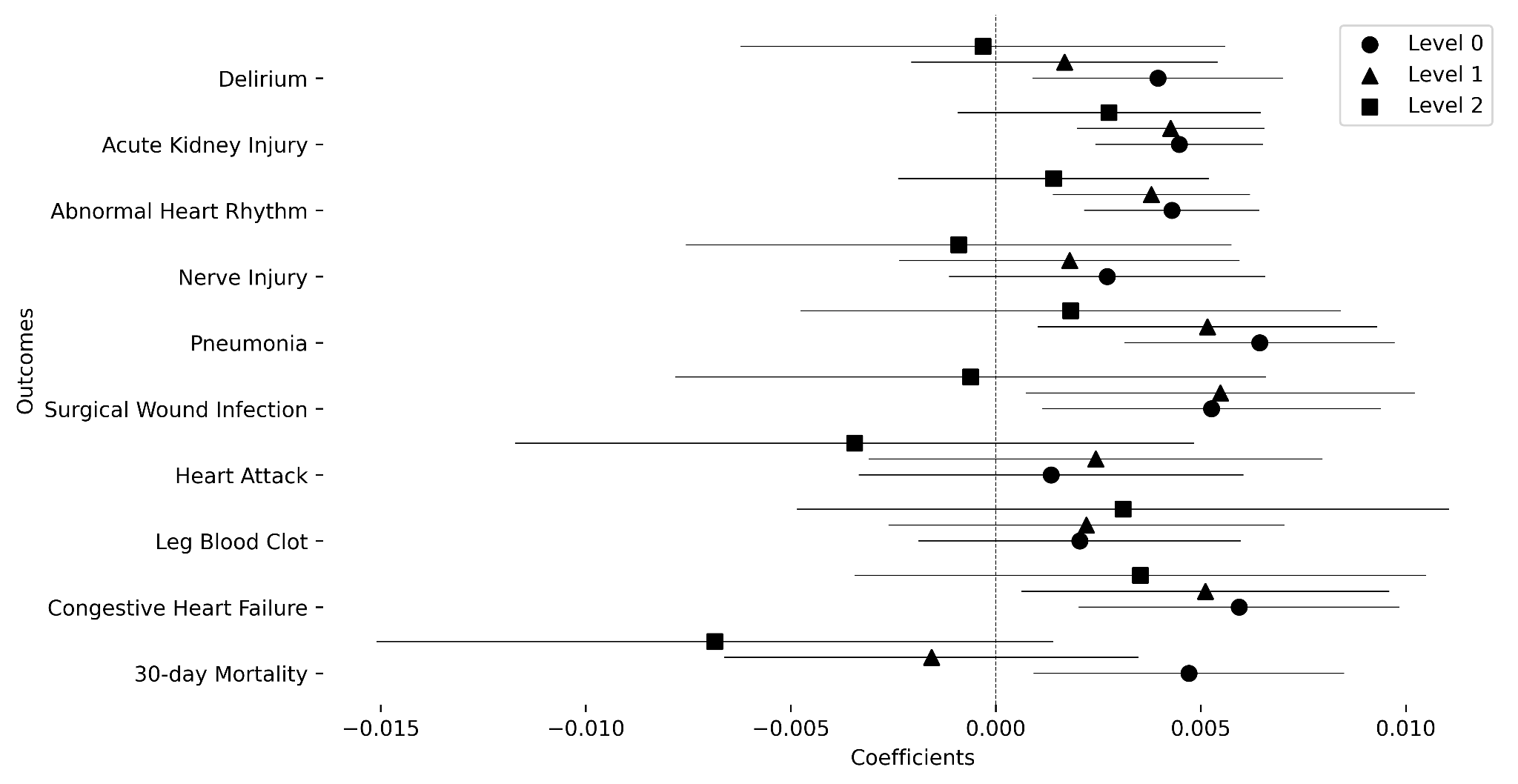


**Supplementary Figure 2**: **Coefficients of the overall ADI value in the association models for each outcome:** Results of each level are plotted with uncorrected 95% confidence intervals


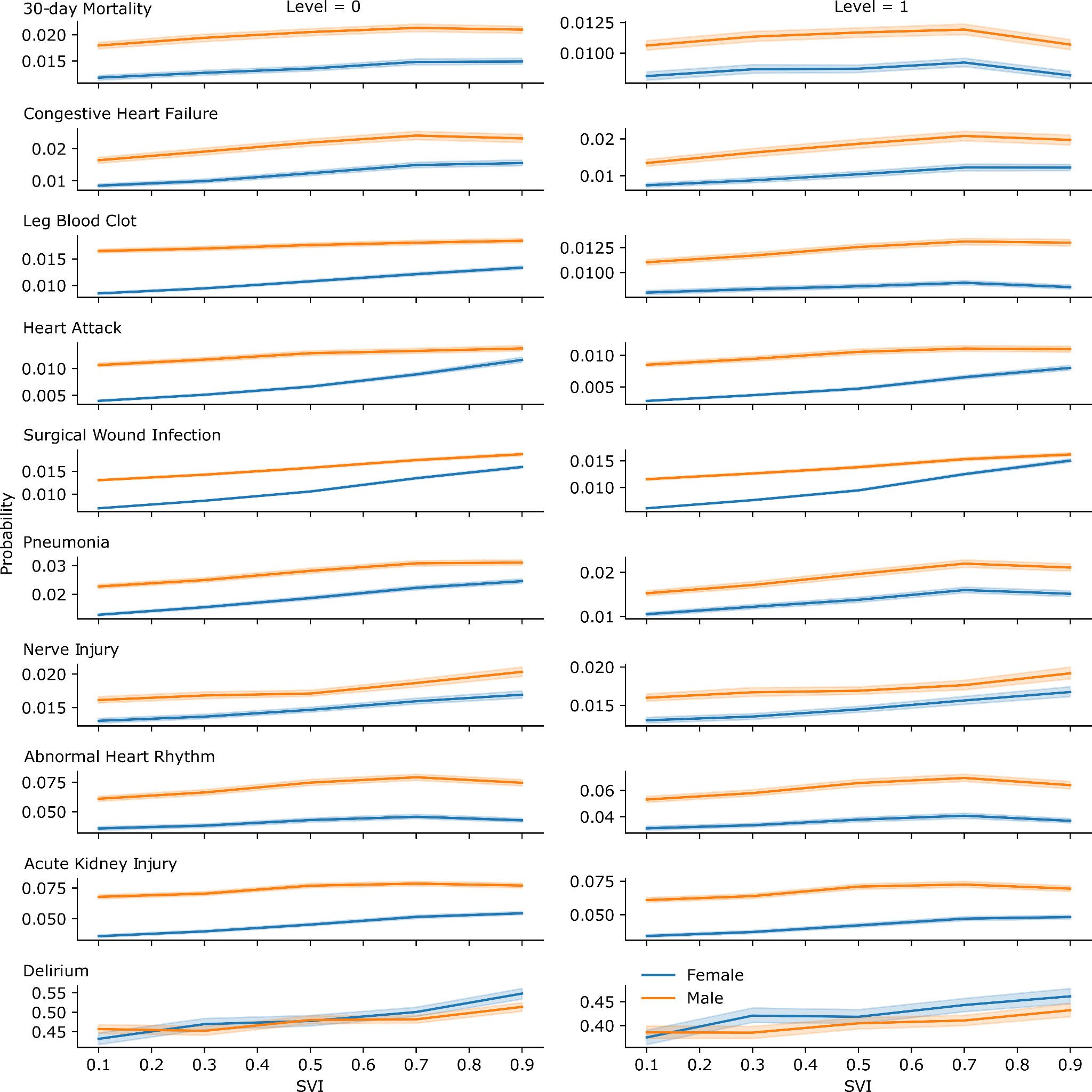


**Supplementary Figure 3**: **Probability of occurrence of postsurgical complications by SVI and sex:** Each column shows a different level of modeling. SVI effect was quantized across quintiles. Shaded region represents the 95% confidence intervals.


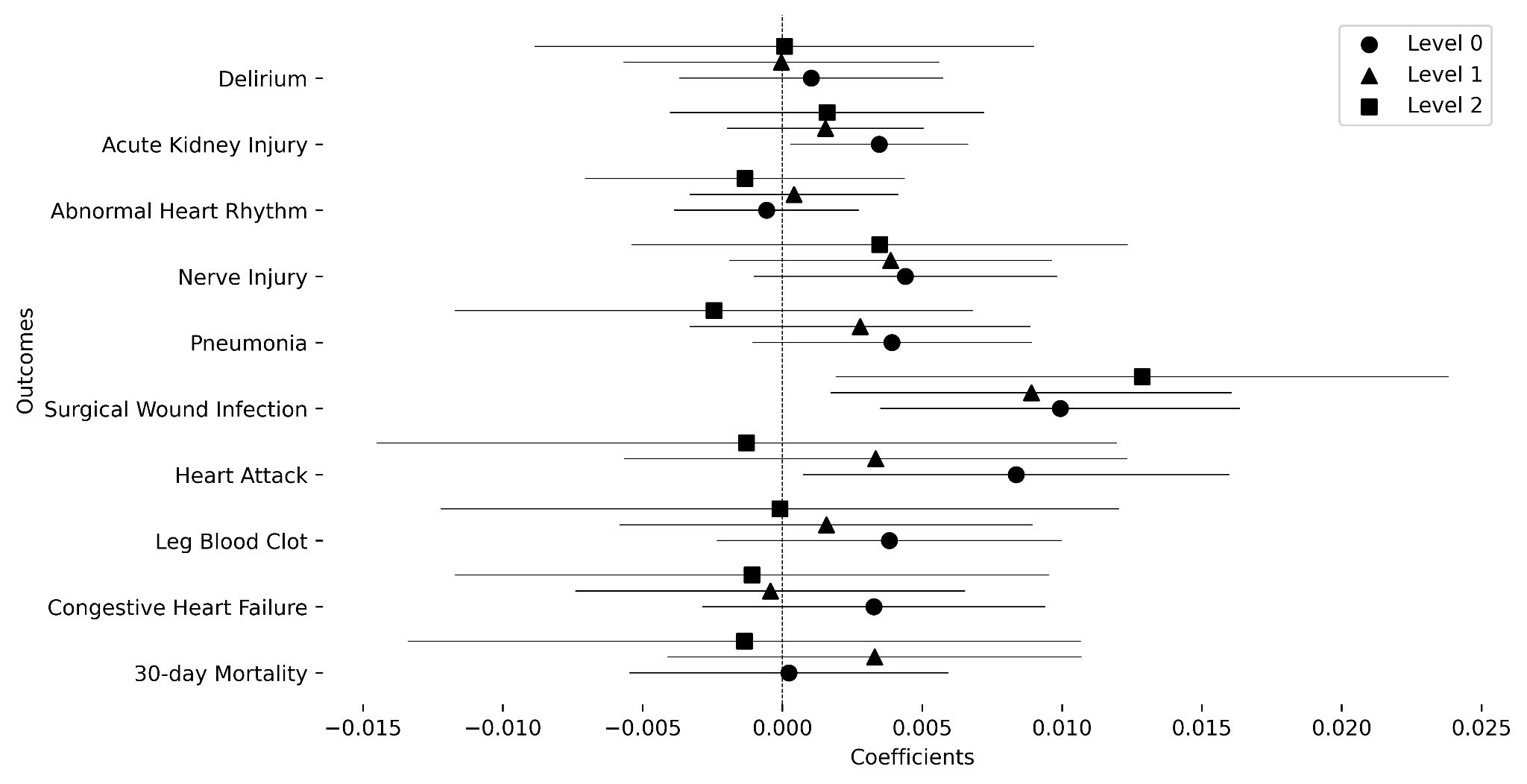


**Supplementary Figure 4**: **Coefficients of the interaction of overall ADI value with sex in the association models for each outcome:** Results of each level are plotted with uncorrected 95% confidence intervals.


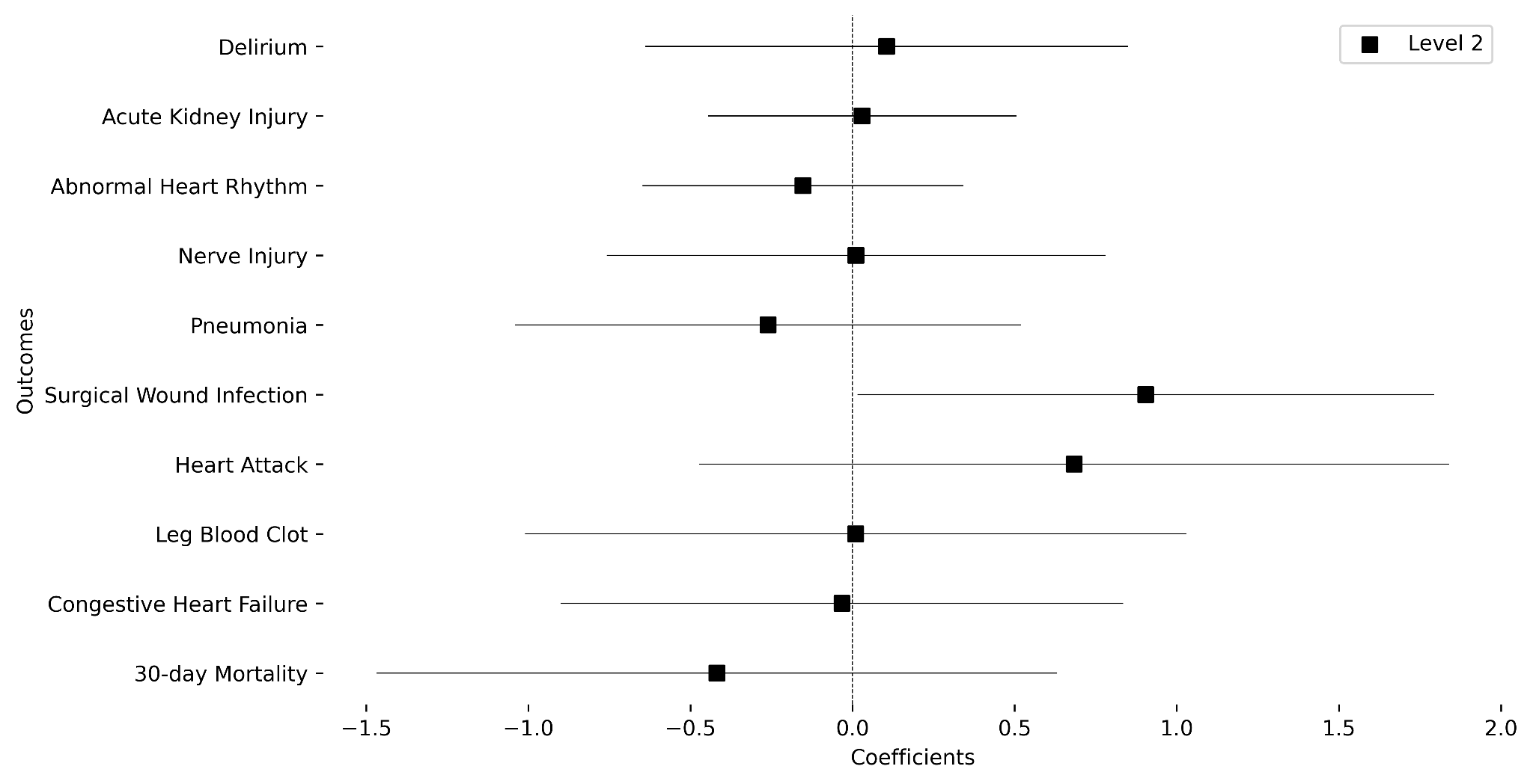


**Supplementary Figure 5: Coefficients of the interaction of overall SVI value with sex in the association models for each outcome for level 2 models:** Results of each level are plotted with uncorrected 95% confidence intervals.


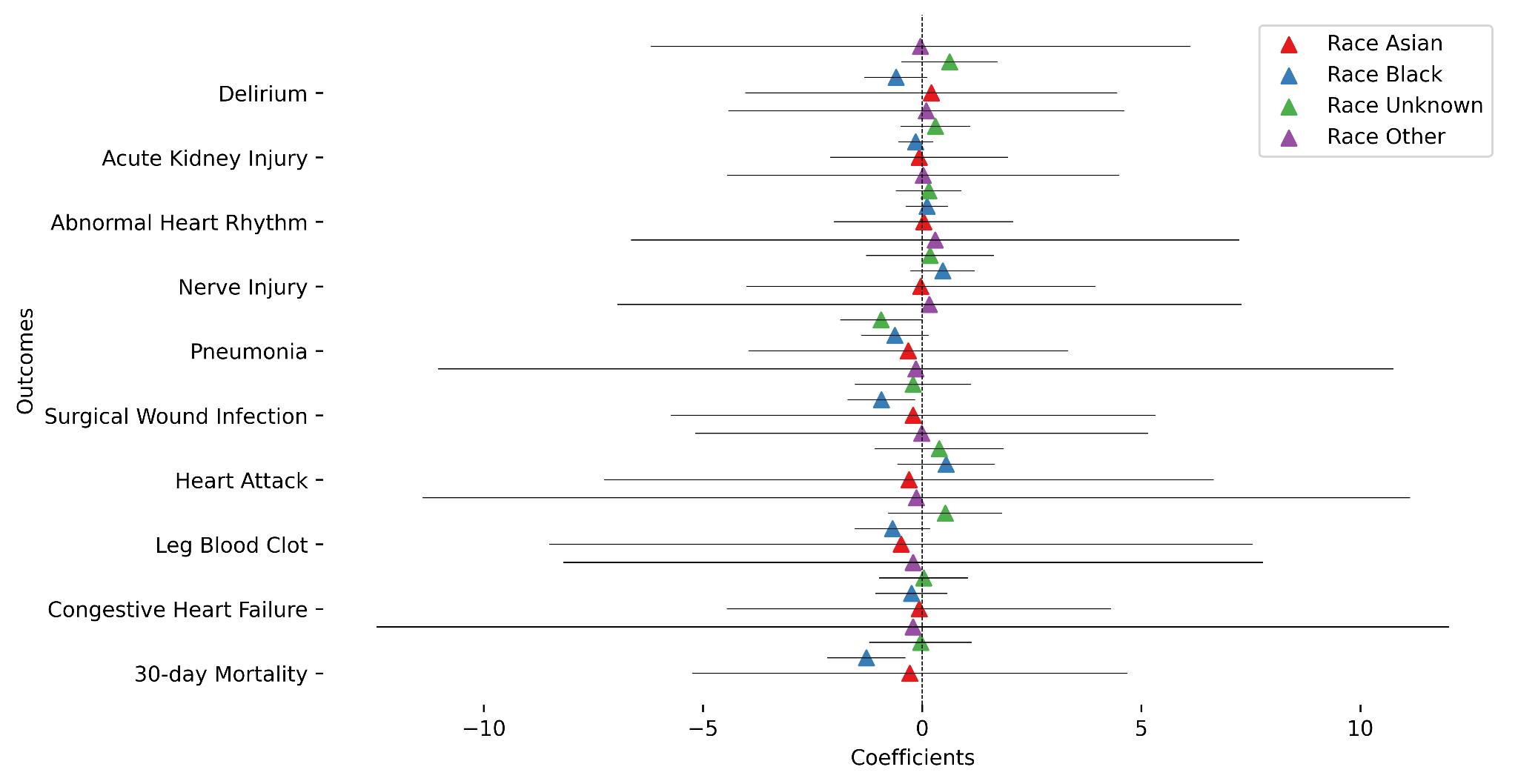


**Supplementary Figure 6**: **Coefficients of the interaction of overall SVI value with race in the association models for each outcome:** Results of each level are plotted with uncorrected 95% confidence intervals.


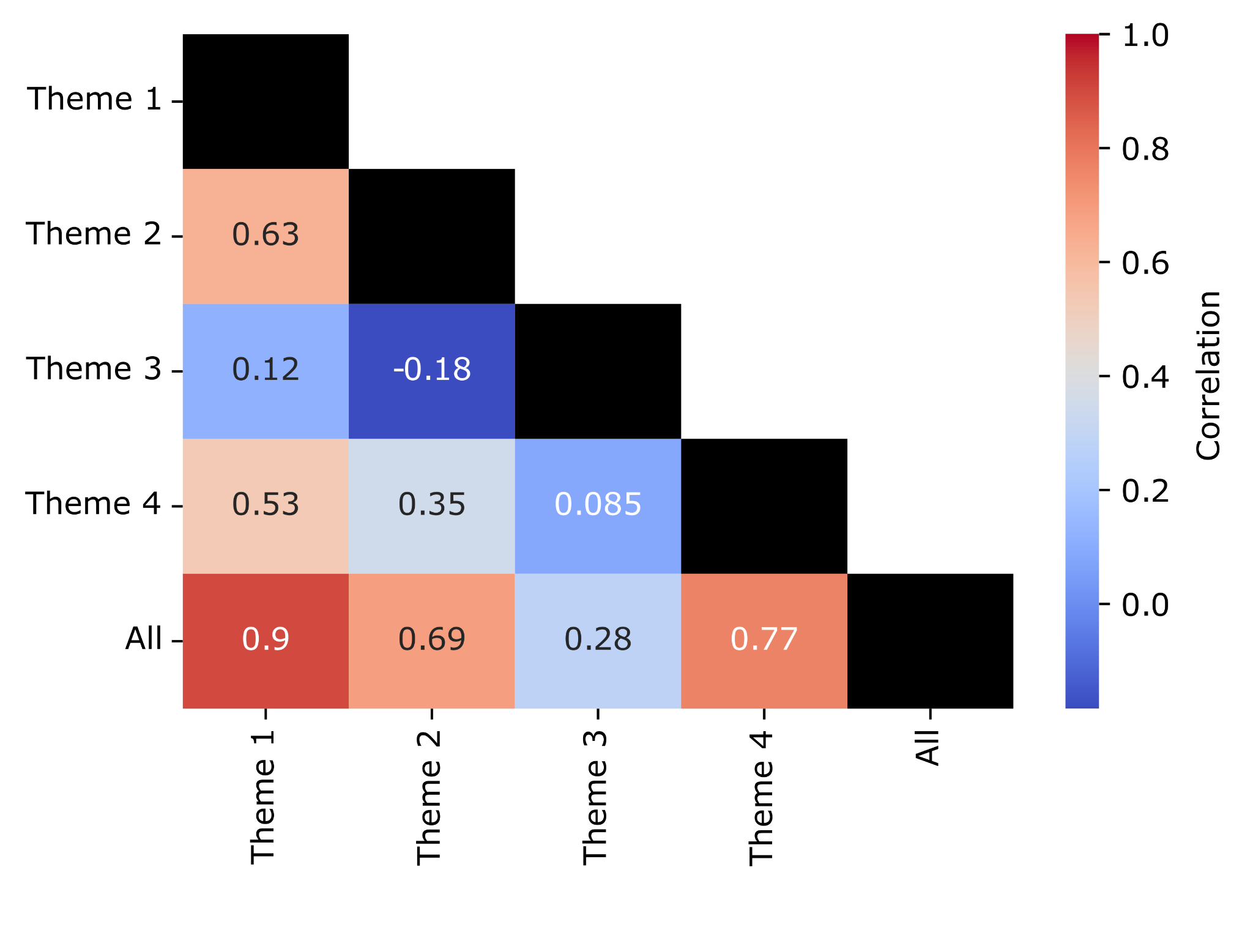


**Supplementary Figure 7: Pearson’s correlation matrix between theme-based SVI values and overall SVI.** These are used to show the contribution of different SVIs to the overall index value and how they correlate.
